# Pharmacological adjuncts to stop bleeding: options and effectiveness

**DOI:** 10.1007/s00068-015-0613-x

**Published:** 2015-12-11

**Authors:** M. Panteli, I. Pountos, P. V. Giannoudis

**Affiliations:** Academic Department of Trauma and Orthopaedics, School of Medicine, University of Leeds, Clarendon Wing, Level A, Great George Street, Leeds, West Yorkshire LS1 3EX UK; NIHR Leeds Biomedical Research Unit, Chapel Allerton Hospital, Leeds, UK

**Keywords:** Bleeding, Trauma, Coagulopathy, Haemostasis, Tranexamic acid, Antifibrinolytics

## Abstract

Severe trauma and massive haemorrhage represent the leading cause of death and disability in patients under the age of 45 years in the developed world. Even though much advancement has been made in our understanding of the pathophysiology and management of trauma, outcomes from massive haemorrhage remain poor. This can be partially explained by the development of coagulopathy, acidosis and hypothermia, a pathological process collectively known as the “lethal triad” of trauma. A number of pharmacological adjuncts have been utilised to stop bleeding, with a wide variation in the safety and efficacy profiles. Antifibrinolytic agents in particular, act by inhibiting the conversion of plasminogen to plasmin, therefore decreasing the degree of fibrinolysis. Tranexamic acid, the most commonly used antifibrinolytic agent, has been successfully incorporated into most trauma management protocols effectively reducing mortality and morbidity following trauma. In this review, we discuss the current literature with regard to the management of haemorrhage following trauma, with a special reference to the use of pharmacological adjuncts. Novel insights, concepts and treatment modalities are also discussed.

## Introduction

Severe trauma and massive haemorrhage represent the leading cause of death and disability in patients under the age of 45 years in the developed world [1–5]. Because of the disproportionate incidence of trauma in younger people, the burden to society is even bigger. Haemorrhage in the setting of trauma is responsible for 30–40 % of trauma mortality [5, 6], with 33–56 % of these deaths occurring in the pre-hospital period [5, 6], whilst one-third of these deaths occur in-hospital [7, 8]. Additionally, haemorrhage represents an important contributory factor for increased mortality of a different aetiology, particularly multi-organ failure and head injury [7, 9].

During the last decades, many advancements have been made in our understanding of the pathophysiology of trauma, the availability of diagnostic adjuncts and the introduction of new resuscitation protocols [10]. Nevertheless, outcomes from massive haemorrhage remain poor, especially in the background of massive blood transfusion and significant coagulopathy [11].

The present review discusses the current literature with regard to the management of haemorrhage following trauma, with a special reference to the use of pharmacological adjuncts. Novel insights, concepts and treatment modalities are also discussed.

## Pathophysiology of haemorrhage

The haemodynamic response triggered by trauma is biphasic, with the systemic arterial pressure initially maintained, followed by a sudden decrease in the systemic arterial pressure with increasing blood loss [12]. If the resuscitation is not adequate, this haemodynamic redistribution can compromise the blood flow to vital organs, therefore further increasing mortality and morbidity [12].

Another significant factor contributing to increased mortality and morbidity following trauma is that of acute coagulopathy [11, 13, 14]. The development of coagulopathy is multifactorial and if not rapidly reversed, it results in a vicious circle often referred to as “acute traumatic coagulopathy” or “acute coagulopathy of trauma shock” [11]. It is usually a combination of the inadequate tissue perfusion because of the resulting hypovolemia and the subsequent cell hypoxia, anaerobic respiration and metabolic acidosis, the thrombin–thrombomodulin-complex generation resulting from the tissue injury and the activation of anticoagulant and fibrinolytic pathways [6, 14]. The resulting state of hyperfibrinolysis is believed to play a central role in the initial coagulopathy, since the maintenance of clot stability is equally important in clot formation [13]. Moreover, the associated hypothermia, which is very common in the traumatised and especially unconscious/sedated patient, leads to a reduced clotting enzymatic activity and a decreased platelet function, a process that further impairs coagulation [6].

Coagulopathy, acidosis and hypothermia are collectively known as the “lethal triad” of trauma and their role in increasing trauma mortality and morbidity is well established [6, 9, 11]. Understanding the pathophysiology of the lethal triad, as well as the importance of its rapid recognition, should serve as the cornerstone for the management of the bleeding trauma patient.

## Blood management in trauma

Failure to promptly recognise bleeding and commence appropriate early management is the leading cause of preventable death following trauma [15]. A multidisciplinary approach aiming to the prompt recognition of shock due to haemorrhage following trauma (defined as loss of 20 % or more of the total blood loss) is of paramount importance, preferentially before the decompensation. The correct management, targeting at preventing or reversing any associated metabolic derangement, should be followed by timely interventions to control bleeding, restore tissue perfusion and achieve haemodynamic stability (Table 1) [5, 9, 14, 16].

**Table 1:** Principles of blood management in trauma

1.	Adherence to Advanced Trauma Life Support (ATLS^®^) principles
2.	Rapid identification of the source of bleeding
3.	Use of pelvic binders and tourniquets as indicated
4.	Surgical exploration, packing, angiographic embolization and stabilisation of pelvic ring in the haemodynamically unstable patient
5.	Correction of post-traumatic coagulopathy
6.	Use of damage control surgery in severe trauma
7.	Fluid resuscitation and transfusion of blood products as indicated

In the pre-hospital setting, the adherence to the fundamental principles of Advanced Trauma Life Support (ATLS^®^) is emphasised, including the rapid transportation of patients to the hospital and limiting therapy to what is necessary to maintain adequate tissue perfusion, such as use of tourniquets to stop life threatening bleeding [14].

In-hospital, provided that ATLS^®^ principles are adhered to, the source of bleeding should be rapidly identified. Multi-sliced computed tomography and ultrasound scans can be used as adjuncts to the clinical examination in the haemodynamically stable patient [14]. In the haemodynamically unstable patient, early control of significant bleeding in the abdomen and/or pelvis can be managed with surgical exploration, packing, angiographic embolisation and stabilisation of pelvic ring [14, 16]. Moreover, serial measurements of serum lactate and base deficit measurements can be used for estimating/monitoring the extent of bleeding and shock, whereas post-traumatic coagulopathy should be monitored and promptly corrected [14]. Additionally, techniques/modalities which have been proved effective for resuscitating the trauma patients include: permissive hypotension (targeting a systolic blood pressure of 80–90 mmHg, until any major bleeding has been stopped) and maintenance of minimally acceptable perfusion to prevent vasoconstriction, dislodgement of the early thrombus and therefore increase of blood loss [4, 5, 14]; minimising the use of crystalloid intravenous fluids during resuscitation, therefore minimising the effect of clotting factors dilution [4, 5, 14]; the use of crystalloid solutions with neutral pH, avoiding hyperchloremic metabolic acidosis associated with resuscitation with large-volume normal saline solutions [4, 5]; the use of blood products including packed red blood cells (RBC), fresh frozen plasma (FFP) and platelets [4]; aggressive prevention of heat loss using heat blankets, blood warming devices and warm intravenous (IV) fluids [4, 16]; and providing adequate analgesia to minimise movement and surges in blood pressure that can increase bleeding [16]. Most recently, attention has been shifted to the pathophysiology at a cellular level, with less sensitive parameters (systolic blood pressure and response to volume resuscitation) being de-emphasised [4].

In addition, in severe trauma, damage control surgery has been proven to be of vital importance [5]. According to this concept, a staged approach is followed with initial abbreviated surgical interventions to control bleeding, contamination and fracture stabilisation, which followed definitive surgical procedures when the patient conditions are improved [5]. Patients that should be considered for this approach are those in deep haemorrhagic shock with signs of ongoing bleeding and established coagulopathy, as well as patients with severe hypothermia, acidosis, major anatomic injuries that cannot be accessed, need for multiple lengthy procedures or presence of major concomitant injuries [14].

## Transfusion standards in trauma

It has been reported that more than 50 % of polytrauma patients are transfused, whilst in several developed countries over 15 % of these patients receive massive transfusions (variably defined as transfusion of more than 10 units of packed red blood cells or transfusion of more than 50 % of total blood volume in 24 h, or transfusion of more than 5 units in 4 h) [17, 18]. Balanced and early use of blood components including red blood cells, platelets, plasma [fresh frozen plasma (FFP) or pathogen-inactivated plasma], fibrinogen and cryoprecipitate has been reported to be effective in major bleeding [14, 19]. In addition, several risk factors have been identified to contribute to an increased blood loss in trauma patients, including use of anticoagulant medications, coagulation disorders, advanced age, use of a pneumatic tourniquet, critical illness and pre-existing anaemia [20–22]. Generally, a haemoglobin (Hb) target between 7 and 9 g/dl is considered to be satisfactory for achieving adequate oxygen delivery to the tissues [14].

Even though the safety of allogeneic blood product transfusion has considerably improved over the last decades, transfusion is still associated with significant morbidity and mortality. Potential hazards of allogenic blood transfusion include the risk of infection (HIV, HBV, HCV) [23–25], non-infectious complications such as ABO incompatibility [26], haemolytic reactions, immunosuppression and even death [27, 28]. Historical transfusion risks such as transfusion errors (incorrect blood component transfused) and other acute transfusion reactions happen very rarely.

A recent meta-analysis on the risks associated with blood transfusion suggests that there is an association between increasing transfusion and mortality, sepsis, multi-organ system dysfunction and acute respiratory distress syndrome (ARDS) [17, 29]. Additionally, a Cochrane review reported that in-hospital mortality of patients receiving restrictive transfusion strategies was reduced, compared to patients managed with liberal transfusion strategies [25]. Therefore, the clinician should always balance the benefits of transfusion against the possible risks, especially in patients who are not unstable, but had haemorrhage. Another facet that should be considered is that blood remains a scarce and costly resource; it has been previously reported that the cost of a unit of red blood cells to the National Health Service in 2011 was £125 [23], whereas this cost is constantly rising.

## Pharmacological strategies for blood management

A wide range of pharmacological agents has been used as adjunct in traditional techniques to obtain haemorrhagic control. These can generally be categorised into two major groups: topical (local) and systemic haemostatic agents.

### Topical haemostatic agents

Topical haemostatic agents are particularly useful to arrest bleeding in areas where the anatomical access is difficult. Such agents include: collagen (acts by triggering platelet aggregation) [14]; gelatin-based products that are often combined with a pro-coagulant substance (swell when in contact with blood reducing blood flow, whereas the pro-coagulant substance enhances haemostasis) [14]; cellulose-based products [14]; fibrin and synthetic glues or adhesives (have both haemostatic and sealant properties) [14]; and tranexamic acid (TXA) applied topically (its use in elective surgery is promising, but it is not yet popularised in trauma patients) [28]. As the use of the above agents is uncommon in the setting of trauma, any further description of their action and properties will exceed the scope of this review. Polysaccharide-based and inorganic haemostatics, on the other hand, have been successfully used in the development of haemostatic dressings, applied mainly in military but also in Civilian situations [6, 14, 30].

#### Haemostatic dressings

Haemostatic dressings include gauze style dressings enriched with powder or granular agents and have successfully been used in the pre-hospital setting, effectively reducing morbidity and mortality through the early control of severe haemorrhage (within 2 min) [6, 13, 30]. The different mechanisms of action include: the concentration of clotting factors at the site of injury; formation of a mucoadhesive seal around the wound (chitosans); and by acting as pro-coagulants (i.e. by activating the coagulation cascade or by providing exogenous clotting factors to the site of injury) [6, 13, 30]. Despite a number of preclinical animal studies and human case series, there is paucity of evidence that could provide an evidence base for using one type of haemostatic dressing over another. However, based on the current literature, Littlejohn et al. suggested that Combat Gauze is the safer and most effective in controlling haemorrhage [13]. Nevertheless, newer agents such as Celox Gauze and ChitoGauze are under investigation and preliminary studies about their use are promising [13].

### Systemic haemostatic agents

The response to extensive tissue injury represents a complex physiological process governed by several regulatory mechanisms, with haemostasis playing an important part of this process. As part of this response, the equilibrium is shifted and clot degradation (fibrinolysis) is stimulated, which in some cases may become pathological (hyper-fibrinolysis) [31], an action that is considered to contribute to bleeding and coagulopathy [32]. Antifibrinolytic agents act by inhibiting the conversion of plasminogen to plasmin, therefore decreasing the degree of fibrinolysis both in patients with normal or exaggerated fibrinolytic responses to tissue injury [28, 31]. The agents most commonly used include TXA, ε-aminocaproic acid and aprotinin.

#### Tranexamic acid

Tranexamic acid (TXA) is a synthetic lysine derivative, that acts as a competitive inhibitor of plasminogen activation, whereas at higher concentrations it acts as a non-competitive inhibitor of plasmin [33]. It is believed that its main action is to improve coagulation trough stabilising the clot [19], but it has been reported that it also acts by inhibiting plasmin-induced platelet activation, therefore preserving platelet function [28]. It has also been suggested that TXA may also act by reducing a broad spectrum of pro-inflammatory effects of plasmin that may be associated to multi-organ system failure [19, 32, 34]. Another potential mechanism of TXA is that by reducing bleeding, it reduces the oxygen demand to the myocardium, simultaneously increasing oxygen supply [35].

Cumulative meta-analysis of the intravenous administration of TXA shows reliable evidence that it significantly reduces the need for transfusion by 38 % in cardiac, orthopaedic, cranial and orthognathic, hepatic, and urological surgery [23]. Yet, some uncertainties and concerns about the effect of TXA on thromboembolic events and mortality are still present [23].

In the largest randomised controlled trial to date (CRASH-2), 20,211 patients with on-going significant haemorrhage or risk of significant haemorrhage were randomised to receive TXA (1 g of TXA infused over 10 min, followed by an intravenous infusion of 1 g over 8 h) or placebo (0.9 % normal saline) [31]. Their results suggest that TXA reduced all-cause mortality (relative risk 0.91, 95 % CI 0·85–0·97) and risk of death because of bleeding (relative risk 0.85, 95 % CI 0.76–0.96) [31]. At the same time, no increased risk of non-fatal vascular occlusive events was recorded [31].

A further analysis of data from the same trial (CRASH-2), combined with data from the UK Trauma and Audit Research Network, investigated the deaths that could be averted according to risk of mortality [35]. The authors suggested that by administrating TXA within 3 h from the traumatic event, the odds of death from bleeding was reduced by about 30 %, whereas the odds of thrombotic events was also reduced by about 30 %. [35]. Thereafter, they recommend that TXA should not be restricted to the most severely injured, but can be safely administered to a wide spectrum of patients with traumatic bleeding [35].

Nevertheless, an unexpected observation from the CRASH-2 trial was an increase in mortality because of bleeding in patients where TXA was administered more than 3 h after the injury; however, an increase in the all-cause mortality was not evident in the same group of patients [31]. Even though this observation is not easy to interpret, possible explanations may include: the adverse effect of TXA in the established disseminated intravascular coagulation [23, 35]; the development of a pro-thrombotic state [23, 35]; reduced effectiveness of TXA when hypothermia or acidosis is present [23, 35]; the possibility of a different effect of the TXA when administered at different times following trauma [23, 35]; and the fact that these patients may have otherwise died from a non-bleeding cause [35].

Another retrospective study investigating the administration of TXA in combat injuries with active bleeding (MATTERs study) suggested that TXA is safe (not associated with an increased risk of thromboembolic events) and effective (a reduction of 6.5 % in absolute mortality was observed) [19]. The dosing regiment used in this study was an intravenous bolus of 1 g of TXA, repeated as felt indicated by the managing physician [19]. Most importantly, the same group suggested that the effect of TXA was more beneficial in the patients with a higher injury severity, i.e. for the patients where a massive transfusion was deemed necessary (in this group, the absolute mortality was reduced by 13.7 %) [19].

It is also of note that both CRASH-2 and MATTERs studies failed to demonstrate any reduction in the need for transfusion [19, 31], in contrast to studies investigating the effect of TXA in elective surgery [23]. This may well reflect the complex equilibrium between the coagulation, the fibrinolytic cascade and the physiology of trauma. Besides, since severely injured patients treated with TXA have a higher survival chance and also a higher incidence of transfusion compared to patients who die early following trauma, this introduces a “survivor bias” [36].

In addition, an on-going trial (CRASH-3) investigates the safety and efficacy of TXA in patients with significant traumatic brain injury [37]. The investigating group hypothesised that TXA counteracts the effect of tissue plasminogen activator that plays an important role in the process of peri-lesional oedema, by blocking the conversion of plasminogen to plasmin [37]. This hypothesis was based on the results of two previous randomized control trials [38, 39]. The first, the CRASH-2 Intracranial Bleeding Study suggested a reduction in intracranial hemorrhage growth, fewer new focal ischaemic lesions on the CT scan and lower all-cause mortality in the patients receiving TXA [38]. Similarly, the second trial reported a reduction in hemorrhage growth and mortality in patients with intracranial injury that received TXA [39].

With regard to the “ideal” loading and maintenance doses of TXA acid, this remains a subject of debate. In the literature, different regimens have been used by trials ranging from 2.5 to 100 mg/kg for loading doses, and 0.25–4 mg/kg/h delivered over 1–12 h for maintenance doses [31]. However, special attention should be given while administrating moderate or high doses of TXA (more than 24 mg/kg), as a dose-dependent increased seizure incidence has been reported in open cardiac surgery [11, 40]. However, in the setting of trauma, calculations based on the weight of the patient may be time consuming and can potentially add a risk for errors. We therefore recommend the use of a fixed dose that is adequate to inhibit fibrinolysis thus achieving haemostasis, but that is also safe in smaller patients.

#### Pre-hospital use of tranexamic acid

The main aim of pre-hospital care remains the rapid transportation of the bleeding patient to a facility for definitive care within the shortest amount of time, in order to stop bleeding and restore the circulating volume [5]. Some authors have suggested that, especially in the developed countries, TXA should be administered in the pre-hospital environment as this could lead to better outcomes through preventing full activation of fibrinolysis [14, 41, 42]. Besides, TXA can be safely stored in vehicles and can be simply administered to the polytraumatised patient [41, 42].

The above suggestion is based on the results of the two largest studies reporting on early administration of TXA [19, 34]. Following a subgroup analysis on death because of bleeding, the collaborators of CRASH-2 trial reported that treatment within 1 h from injury significantly reduced the risk of death (RR 0.68, 95 % CI 0.57–0.82), whereas treatment given between 1 and 3 h also reduced the risk of death (RR 0·79, 0.64–0.97) [34]. Thereafter, the odds ratio of TXA on death due to bleeding was calculated as low as 0.61 (95 % CI 0.50–0.74), which is multiplied by a factor of 1.15 (95 % CI 1.08–1.23) for every hour that passes from the injury [34]. The MATTERs study group reported similar results, as all patients had the first dose of TXA administered within 1 h from injury [19].

#### ε-Aminocaproic acid

ε-Aminocaproic acid is another synthetic lysine analogue, with a smaller half-life than TXA. The presence of subtle molecular variations between TXA and ε-aminocaproic acid, make ε-aminocaproic acid at least ten times less potent, therefore restricting its use in the cases that TXA is not available [14, 36].

#### Aprotinin

Aprotinin, a serine protease inhibitor, acts by directly inactivating free plasmin, having little effect on bound plasmin [43, 44]. Even though aprotinin was reported to effectively reduce bleeding and transfusion requirements in major surgery with a dose–response profile [44], its marketing was suspended in November 2007 questioning its safety [43]. Recently, the regulatory body has licensed aprotinin only for myocardial revascularisation [43]. Regardless of the presence of previous reports investigating its efficacy in the setting of trauma, this off-label use is now contraindicated [45–47].

#### Recombinant factor VIIa

Recombinant factor VIIa (rFVIIa) is a haemostatic agent approved for bleeding episodes in haemophilia patients [48–50]. Multiple studies have investigated its effectiveness in trauma, reporting decreased blood loss that did not, however, translate to better patient outcomes [48–50]. On the contrast, concerns about adverse thromboembolic events often resulting in serious morbidity and mortality were raised, whereas this off-label use was associated with an additional financial burden for hospitals [48]. Thereafter, the use of rFVIIa is not recommended in trauma patients [48–50].

#### Anticoagulated patients

With the rapid ageing of the population in most of the word, the number of anticoagulated patients is expected to continue growing [51–53]. As a result, the number of trauma patients using anticoagulant or antiplatelet agents is also expected to increase [51–53]. Previous reports suggest that pre-injury use of Warfarin represents an independent risk factor of mortality following trauma through altering bleeding and coagulation mechanism, whereas use of antiplatelet agents is not associated with an increased mortality [51, 53, 54]. Yet, the use of warfarin or antiplatelet agents is associated with an increased risk of intra-cranial haemorrhage [51].

Based on these evidences, rapid treatment protocols may be considered, especially in the presence of intra-cranial head trauma where a prompt diagnosis is of paramount importance [55]. Anticoagulation reversal (INR of 1.5 or less) may include the use of Vitamin K, FFP, administration of prothrombin complex concentrates (contain a combination of factors II, VII, IX and X) [51, 55, 56], or a combination of the above that can be more effective [52]. Where there is a necessity of reversing the effects of antiplatelet agents, 10 units of platelets and/or desmopressin have been reported as effective [14, 57].

## Overview

Severe trauma and massive haemorrhage remains the leading cause of death and disability in the young population. Even though much advancement has been made in the management of these patients with the introduction of complex resuscitation protocols, the outcomes remain poor. The use of anti-fibrinolytic agents is one of the most promising interventions, but their benefits should be balanced with the risk of complications, especially thrombosis. Based on the available evidence and our experience, our recommendations are outlined in Table 2.

**Table 2:** Recommendations for use of pharmacological adjuncts

		Administration/dose
Topical haemostatic agents		
Indications	To arrest bleeding in areas where the anatomical access is difficult	
Recommendations	Combat Gauze, Z-Medica, Wallingford, CT	Pack gauze into wound and apply manual pressure for 3 min
Systemic haemostatic agents		
Indications	To arrest heavy bleeding that cannot be controlled with direct pressure or application of topical haemostatic agents	
Recommendations	Tranexamic acid (TXA), Pfizer	Dose: 1 g infused over 10 min, repeated as indicated Use within 3 h from the traumatic event, but preferably within 1 h Pre-hospital use is advocated TXA should be included in every trauma resuscitation protocol

Following the publication of the CRASH-2 trial results, TXA has been incorporated into most trauma management protocols worldwide, both in the pre-hospital and hospital environment. The World Health Organization (WHO) has also included TXA into the List of Essential Medicines, whereas the National Institute for Health and Care Excellence (NICE) is expected to publish a Clinical Guideline on the assessment and management of major trauma in February 2016 including the off-label use of TXA [58, 59].

The benefits of this easy-to-use molecule are also likely to be greater in the elderly population who have a higher baseline risk of death from haemorrhage and thromboembolic events, an observation based on the reduction of the arterial events in this patient group [35]. Pererl et al. have developed and validated a prognostic model for risk of mortality based on clinical parameters such as age, Glasgow Coma Score (GCS), systolic blood pressure and geographic area [8]. This protocol could potentially be used as an additional tool for deciding whether or not to use lifesaving interventions such as TXA. In any case, the treating physician should use all the available information and their clinical judgment to the best interest of their patient.

Finally, it has been estimated that every year up to 128,000 deaths worldwide can be averted if TXA is administered within 1 h from injury, whereas when administered within 3 h, up to 112,000 deaths can be averted [60]. This potential is more significant in low and middle income countries [60], whereas TXA is likely to be highly cost-effective in any income setting as reflected by the life years saved [61].

## Future directions

Pre-hospital care should be improved with TXA been incorporated in the management of bleeding trauma patients, whereas advanced trauma resuscitation protocols should be in place in order to reduce time to definitive haemorrhage control. Future research should aim into investigating trauma coagulopathy and the effect of interventions such as that of TXA. Finally, further pharmacokinetic and pharmacodynamic studies are required to clarify the exact mechanism of action of TXA, as well as the optimal doses in both the adult and paediatric populations.
